# Osteosarcoma in Pediatric Patients and Young Adults: A Single Institution Retrospective Review of Presentation, Therapy, and Outcome

**DOI:** 10.1155/2014/402509

**Published:** 2014-04-30

**Authors:** Candace L. Haddox, Gang Han, Leon Anijar, Odion Binitie, G. Douglas Letson, Marilyn M. Bui, Damon R. Reed

**Affiliations:** ^1^Morsani College of Medicine, University of South Florida, Tampa, FL 33612, USA; ^2^Department of Biostatistics, School of Public Health, Yale University, New Haven, CT 06510, USA; ^3^Sarcoma Program, H. Lee Moffitt Cancer Center and Research Institute, 12902 Magnolia Drive, Tampa, FL 33612, USA; ^4^Adolescent and Young Adult Program, H. Lee Moffitt Cancer Center and Research Institute, 12902 Magnolia Drive, Tampa, FL 33612, USA; ^5^Anatomic Pathology Department, H. Lee Moffitt Cancer Center and Research Institute, 12902 Magnolia Drive, Tampa, FL 33612, USA

## Abstract

*Background*. Little is known about how cumulative chemotherapy delivery influences the poorer outcome observed in young adult (YA, 18–40 years) versus pediatric (<18 years) osteosarcoma patients. Here, we retrospectively examined differences in presentation, therapy, including cumulative chemotherapy dose, and outcome in YA and pediatric patients. *Methods*. We reviewed 111 cases of high-grade osteosarcoma at Moffitt Cancer Center between 1988 and 2012. Presentation factors, therapies, and survival were compared between YA and pediatric cohorts. *Results*. The cohorts were equivalent with respect to metastatic status, gender, tumor size, tumor site, and histological subtype. We found that the YA patients tended to have poorer histologic response to neoadjuvant chemotherapy measured by necrosis with 55% and 35% of pediatric versus YA patients responding favorably (*P* = 0.06). Only 39% of YA patients achieved the typical pediatric dose of methotrexate, doxorubicin, and cisplatin. These patients had a 3-year EFS of 76% (CI 53–100%) versus 47% (CI 26–69%; *P* = 0.09) in those who received less chemotherapy. *Conclusion*. Age continues to be a prognostic factor in osteosarcoma. Our study suggests that presentation factors are not associated with prognosis, while poorer response to chemotherapy and lower cumulative dose of chemotherapy delivered to YA patients may contribute to poorer outcomes.

## 1. Introduction

Osteosarcoma is the most common primary tumor of bone in patients under the age of 40 years. Roughly 1,000 new cases are diagnosed each year, with 400 of these diagnosed in pediatric patients under 18 years [1]. Before the 1970s, amputation was the main therapeutic modality and was associated with a 5-year survival of less than 20% [2]. Several landmark studies demonstrated improved outcomes with the addition of neoadjuvant and adjuvant chemotherapy, and long-term survival for localized patients now approaches 70% [3–7].

More recent advances have optimized surgical approaches such as limb salvage procedures, rendering amputation a rarity [8–10]. Additionally, large cooperative groups have successfully completed international clinical trials, leading to improved standardization for the treatment of osteosarcoma [11]. For localized pediatric osteosarcoma, high-dose methotrexate, doxorubicin, and cisplatin make up the standard backbone of chemotherapy on cooperative group trials. For young adult osteosarcoma patients, less is known about how treatment and disease biology influence survival; however, many studies have shown age to be a prognostic factor for osteosarcoma and other cancers [4, 12–16].

Young adult (YA) patients, defined in this study as patients aged 18–40 years at diagnosis, have been underrepresented in clinical trials for many cancers, including osteosarcoma [17]. YA patients are not typically treated in pediatric centers in our area. These patients represent 7% of new cancer diagnoses in the United States [18]. With few exceptions, YA patients have a worse prognosis than pediatric and older adult populations for a given histology [18]. That is, younger breast and colon cancer patients have a poorer outcome than older patients, and adult patients with traditionally pediatric diagnoses such as acute lymphoblastic leukemia also fare worse than their pediatric counterparts [19]. This may be due to presentation factors, such as late presentation due to patient denial, primary care physicians having a low suspicion of malignancy in a young adult, or inadequate access to care. Therapy factors such as poor clinical trial participation, lack of a care system focused on the needs of the YA patient, and poor adherence to therapy may also contribute to the overall worse outcome [20–24]. It is known that adherence to a planned chemotherapy regimen impacts survival, and modifying the regimen in osteosarcoma has been associated with poorer local recurrence-free survival [25]. Finally, it is also possible that disease and host factors play a role, such as tolerance of and response to therapy and distinct tumor and host biology. A single institution study found that YA patients with rhabdomyosarcoma have a higher stage at diagnosis and higher risk histology contributing to an overall poorer outcome [26].

Here, we retrospectively compared YA patients (diagnosed at 18–40 years of age) versus pediatric patients (under 18 years old) in terms of presentation, therapies received, and outcome in order to determine which factors are associated with the poorer outcomes observed in the YA population. We provide novel analysis of the impact of total chemotherapy delivered in YA patients in terms of cumulative dose on event-free survival, overall survival, and patterns of recurrence.

## 2.  Patients and Methods

### 2.1. Patients

A retrospective review was conducted of all osteosarcoma patients treated at Moffitt Cancer Center between 1988 and 2012. After formal institutional review board approval, 111 charts were accessed for review. Inclusion criteria included diagnosis of high-grade osteosarcoma by pathology. The diagnoses were further confirmed by the study pathologist (MMB). Exclusion criteria included tumors involving jaw and skull and presenting to our institution for therapy following recurrence. Data collected included age at diagnosis, gender, location of primary disease, tumor size, histological subtype, presence of metastatic disease at diagnosis, pathologic fracture, surgical approach, prosthesis placement, margin status, percent-tumor-necrosis at resection, intensity of chemotherapy, treatment with radiation therapy, timing and location of recurrence, amount of disease at recurrence, follow-up time, and survival.

### 2.2. Chemotherapy

We further researched the chemotherapy delivered to all patients and collected data on specific agents, number of doses, and cumulative dose in units/m^2^ delivered to the YA patients. The pediatric patients received chemotherapy at 5 different institutions; consequently, records detailing systemic therapy were incomplete and not analyzed. For pediatric patients who did have complete records at our institution, we found that 94% (17 of 18 patients) had completed planned therapy according to protocol. Through personal communication, we confirmed that all institutions treated osteosarcoma patients on or as per cooperative group studies as opposed to an individualized or institutional protocol, and patients were assumed to receive complete therapy as has been done in previous studies. At the time of diagnoses for our patients, there were 3 available protocols (POG9351/CCG7921, P9754, and AOST0331), which incorporated a cumulative dose of 120–144 g/m^2^ of methotrexate, a minimum of 450 mg/m^2^ of doxorubicin, and 480 mg/m^2^ of cisplatin, with some patients receiving ifosfamide, ifosfamide and etoposide, and/or L-MTP-PE on POG9351/CCG7921, and possibly PEG-interferon alfa-2b on AOST0331. For YA patients to be categorized as achieving the MAP regimen overall (MAP+), patients must not have missed more than 2 doses of methotrexate, 1 dose of doxorubicin, or 1 dose of cisplatin. All others were considered MAP−. For YA patients with recurrence (local recurrence or identification of disease at a different site that was not present at the time of diagnosis) or progression on therapy (enlarging primary tumor or new tumor(s) not present at the time of diagnosis), we also collected data on second-line therapies used and postrelapse survival.

We analyzed percent necrosis as a categorical variable based on the Huvos Grading System [27]. Percentage of necrosis greater than 90% following neoadjuvant chemotherapy was considered “good” and less than 90% was considered “poor.” Patients who did not receive neoadjuvant chemotherapy or had missing chemotherapy records were removed from the analysis.

### 2.3. Statistical Analysis

Descriptive statistics of the patient characteristics were computed for both cohorts and tested with Fisher's exact test for discrete variables and Wilcoxon rank sum test for continuous variables. Doses of chemotherapy given to the YA cohort were compared to the pediatric typical doses for each agent (11 or 12 doses for methotrexate, 6 doses for doxorubicin, and 4 doses for cisplatin). The proportion of patients achieving pediatric doses (MAP+) for each agent was estimated based on the binomial distribution, and the corresponding variance was computed based on normal approximation. Survival was computed from the time of tissue diagnosis by biopsy until death. Postrelapse survival was computed from the date of recurrence until death. Kaplan-Meier's product limit approach and the log-rank test were used to estimate the survival probability and compare the patient survival from different groups, respectively. Analyses were conducted using the statistical software Minitab 16 (Minitab Inc., State College, PA) and SAS 9.3 (SAS Institute Inc., Cary, NC).

## 3. Results

**Figure 1:**
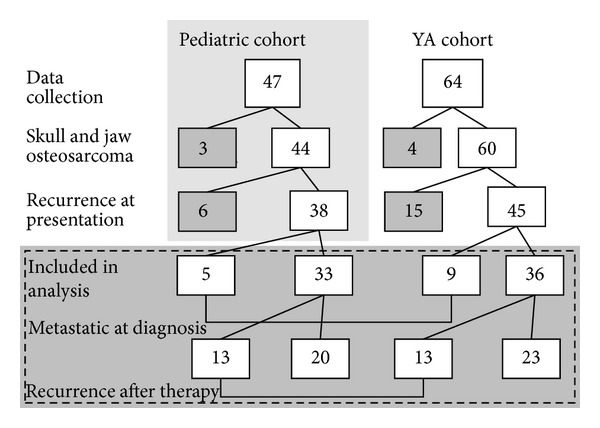
Patient cohorts (pediatric and YA). Of the 83 patients included in our analysis, 14 had metastatic disease at diagnosis, and 26 patients went on to have recurrence.

Of the 111 patients with high-grade osteosarcoma, 47 patients were children (pediatric cohort, <18 years at diagnosis) and 64 patients were young adults (YA cohort, 18–40 years at diagnosis). We excluded 7 patients with skull and jaw osteosarcoma (3 pediatric and 4 YA) as these patients are specifically excluded from COG protocols and may have a different clinical course [28–31]. We also excluded 21 patients who first presented to Moffitt with recurrent disease (6 pediatric and 15 YA). The 83 patients for analysis included 45 YA patients and 38 pediatric patients (Figure 1). The median follow-up time was 3.2 years for the pediatric cohort and 2.4 years for the YA cohort (*P* = 0.37).

The relative number of metastatic patients did not differ significantly between the two cohorts. Five patients (13%) in the pediatric cohort and 9 patients (20%) in the YA cohort had metastatic disease at diagnosis (*P* = 0.57). These patients were excluded in the therapy and outcome analyses, leaving 33 pediatric patients and 36 YA patients.  Table 1 summarizes the presenting characteristics for each cohort. The median ages at diagnosis for the pediatric and YA cohorts were 15 (IQR 14–16) and 23 (IQR 19–26), respectively (*P* < 0.001). There was not a statistically significant difference between the two cohorts in terms of gender (both had male predominance), tumor size, or presence of pathological fracture. We observed a higher number of YA patients with disease in sites other than the long bones, such as the pelvis (*n* = 3), hand (*n* = 1), clavicle (*n* = 1), rib (*n* = 1), and spine (*n* = 1); however this was not significant (*P* = 0.12). Overall, histologic subtype was not significantly different between the two cohorts; notably, however, giant cell osteosarcoma was only seen in the YA cohort (*n* = 2) and telangiectatic osteosarcoma was only seen in the pediatric cohort (*n* = 2) (Table 1).

In terms of therapies received, 100% of the pediatric patients and 94% of the YA patients underwent surgery, all patients received chemotherapy, and 12% and 14% of patients in the pediatric and YA cohort, respectively, also received radiation therapy (Table 2). The 2 YA patients who did not receive surgery were deemed to be poor surgical candidates due to tumor location or had progression on chemotherapy. One patient in each cohort had positive margins following primary surgical resection: the pediatric patient received adjuvant therapy followed by reexcision and the YA patient had a postsurgical period complicated by poor wound healing and subsequently began adjuvant chemotherapy and developed metastatic disease while on therapy. All four patients in the pediatric cohort who received radiation therapy were being treated for recurrence of disease. In the YA cohort, two of the patients underwent radiation as part of their primary therapy and three received radiation following recurrence.

**Figure 2:**
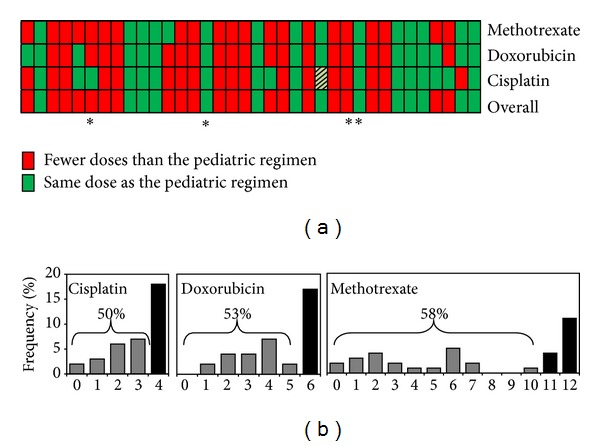
The YA cohort received less chemotherapy than the typical pediatric regimen. (a) Heat map for the YA cohort's chemotherapy regimen, with each column representing 1 patient. The green squares indicate that the patient received the typical dose according to pediatric osteosarcoma protocols. The red squares indicate that the patient received fewer doses than the typical pediatric dose. Asterisks (∗) indicate patients who progressed on primary therapy. Light green box indicates patient who received etoposide/ifosfamide instead of cisplatin. (b) Percentage of patients in the YA cohort that received each dose of chemotherapy. The black bars indicate the typical dose of each drug for pediatric patients. The percentages shown indicate the percentage of patients in the YA cohort that received less than the typical dose.

We further characterized the chemotherapy regimen that the YA cohort received and found that this cohort was treated with a variety of chemotherapy agents with variation in cumulative dose (Figure 2). We found that only 39% of the YA cohort completed the standard pediatric MAP regimen overall (MAP+). Agent-specific rates of achieving a pediatric dose were 58% (95% CI 45–87), 53% (95% CI 36–77), and 50% (95% CI 34–73) for methotrexate, doxorubicin, and cisplatin, respectively (Figure 2(a)). Of the 14 YA MAP+ patients, only 35% had good histologic response to chemotherapy compared with 55% in the pediatric cohort (*P* = 0.06).

**Figure 3:**
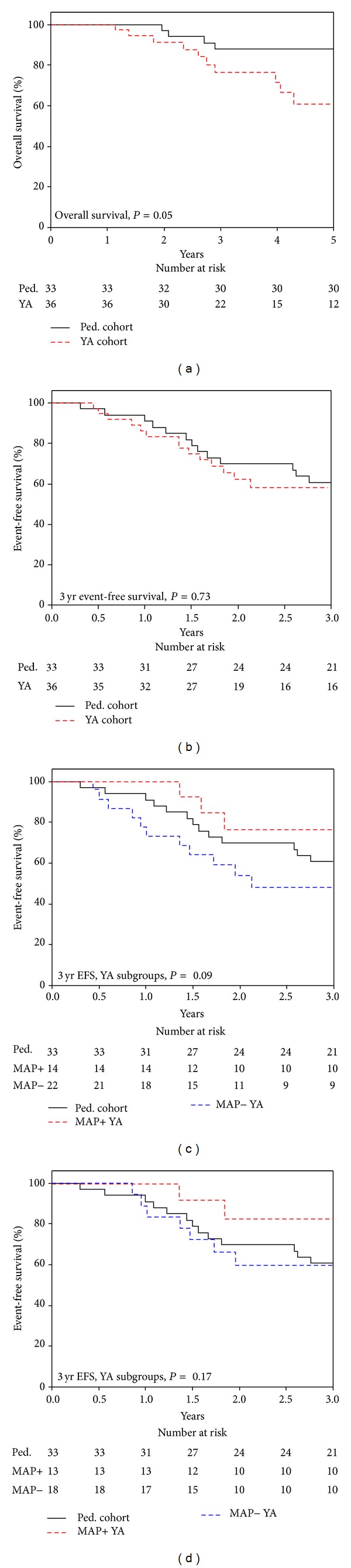
Localized disease outcomes for pediatric and YA cohorts demonstrate better overall survival in pediatric cohort and improved 3-year EFS for MAP+ YA patients. (a) Five-year overall survival of patients with localized disease in the pediatric and YA cohort (*P* = 0.05). (b) Three-year EFS for pediatric cohort and YA cohort (*P* = 0.73). (c) Three-year EFS of YA patients who received less chemotherapy than the typical pediatric regimen (MAP−) compared to YA patients who received the typical pediatric regimen (MAP+) (*P* = 0.09). (d) Three-year EFS of YA MAP− patients compared to YA MAP+ patients. Patients who had recurrence during primary MAP therapy were removed (*P* = 0.17).

The overall survival probability for patients with localized disease at diagnosis was 88% (95% CI 77–99) for the pediatric cohort and 61% (95% CI 41–81) for the YA cohort at 5 years (Figure 3(a); *P* = 0.05). The 3-year event-free survival (EFS) was 60% (95% CI 44–77) and 58% (95% CI 41–75) in the pediatric and YA cohorts, respectively (Figure 3(b); *P* = 0.73). When the YA cohort was stratified based on whether the pediatric regimen of chemotherapy was achieved, the 3-year EFS trended towards being poorer in the MAP− subgroup (Figure 3(c); *P* = 0.09). YA patients who achieved the typical pediatric doses of the MAP regimen (MAP+) had a 3-year EFS of 76% (95% CI 53–100), whereas YA patients who did not achieve the typical pediatric dose (MAP−) had a 3-year EFS of 47% (95% CI 26–69). Four patients who did not complete the pediatric MAP regimen had progression on therapy, prompting a change in chemotherapy regimen, and the analysis was repeated without these patients. When these patients were eliminated, the MAP+ YA patients tended to have better 3-year EFS; however, the trend observed in Figure 3(c) was no longer present (Figure 3(d); *P* = 0.17).

Of the 36 patients in the YA cohort who had localized disease at diagnosis, 13 (36%) had recurrence within 3 years of diagnosis, and only 3 of these patients completed the pediatric MAP regimen (Table 3). For the 13 YA patients with recurrence, the median postrelapse survival was 1.5 years (IQR 1.2–2.2 years). Twelve patients had records available on therapies used following recurrence: 75% of these patients had surgery, 25% had radiation therapy, and 100% received chemotherapy. The chemotherapeutic agents used included ifosfamide (*n* = 7, 58%), Adriamycin (*n* = 5, 42%), cisplatin (*n* = 5, 42%), etoposide (*n* = 5, 42%), and methotrexate (*n* = 1, 8%), as well as other agents (*n* = 6, 50%). Additionally, 25% (3/12) of patients participated in clinical trials at some point after relapse. Lung metastasis was the most common distant recurrence overall and was found in 10 out of 13 patients (77%). Local recurrence was common at first recurrence (*n* = 7, 54%), followed by recurrence at lung (*n* = 5, 38%) and spine (*n* = 1, 8%). Two patients had multifocal first recurrence. Subsequent recurrences included lung, local recurrence, brain, heart, rib, chest wall, and acetabulum.

## 4. Discussion 

Intensive, multiagent chemotherapy maximizes outcome in osteosarcoma patients, and we have found that when pediatric cumulative doses were achieved at our institution, YA outcomes were similar to those in our pediatric cohort. Unfortunately, we found that in the past 15 years, 61% of the YA cohort had received less chemotherapy than is traditionally given to pediatric osteosarcoma patients. Although this study was not powered to detect a significant difference, YA patients who received less chemotherapy tended to have inferior 3-year EFS when compared to pediatric patients. In contrast, patients in the YA cohort who were treated with intensive pediatric chemotherapy at our institution tended to have outcomes similar to the pediatric cohort in terms of EFS. This implies that chemotherapy intensity may contribute to different outcomes in pediatric and YA patients. A recent meta-analysis of several cooperative groups, not including COG, found a higher incidence of mucositis and thrombocytopenia in children than in YA patients [32]. Pediatric patients were also more likely to have greater tumor necrosis, better histologic response following neoadjuvant chemotherapy, and increased overall survival [32]. This may suggest that the pediatric patients had effectively more intense chemotherapy, although the analysis was unable to determine whether it was due to physician acceptance of toxicity, greater dose delivered, or altered pharmacologic effects of equivalent dosing between the two populations. Although the results of our study are consistent with others showing that YA osteosarcoma patients experience worse EFS and overall survival, we believe that this study provides additional detail regarding the potential role of cumulative chemotherapy doses delivered and outcome in the YA population and suggests that efforts should be made to treat YA patients similarly to pediatric patients when possible.

We did not find obvious presentation differences between our cohorts in terms of known clinical prognostic features such as metastatic status. In terms of primary location, we observed fewer tumors in the long growing bones of puberty among YA patients and more pelvic tumors. Although this finding was not significant, pelvic and nonextremity tumors confer a worse prognosis and may have contributed to the poorer outcome of our YA patients [33]. We also found giant cell, extraskeletal, and periosteal osteosarcoma exclusively in the YA patients. While telangiectatic osteosarcoma was exclusively in the pediatric cohort, chondroblastic osteosarcoma was relatively more abundant in the YA cohort.

In a recent children's oncology group (COG) review of osteosarcoma patients enrolled on a protocol with uniform chemotherapy that investigated the relationship between presenting factors and survival, presenting factors such as tumor site and metastatic status did not appear to contribute to the inferior outcomes observed in the osteosarcoma YA population, as our study found [14]. Histologic response to neoadjuvant chemotherapy was not significantly different between pediatric and YA patients; however, YA patients who had poor response were more likely to have inferior outcomes than pediatric patients with poor histologic response. Specific chemotherapy delivery in terms of cumulative dose was not reported or collected in that study. They concluded that differences in tumor biology and chemotherapy metabolism may have contributed to the outcome discrepancy between pediatric and YA patients.

We also made several observations consistent with the hypothesis that the biology of YA osteosarcoma is distinct from pediatric osteosarcoma, potentially explaining the discrepancy in observed outcomes. We showed that our YA patients had poorer overall survival compared to pediatric patients, which is consistent with other studies in osteosarcoma. Interestingly, the 3 year EFS curves were nearly identical between the two cohorts, perhaps suggesting that pediatric patients have a better postrelapse survival than YA patients. This may suggest that YA osteosarcoma is a more aggressive disease or has greater resistance to second-line therapies. Additionally, our data may suggest that YA osteosarcoma has poorer histologic response to neoadjuvant therapy, which is a known prognostic marker for osteosarcoma outcome. The percentage of patients achieving Huvos grade III/IV necrosis (90–100% necrosis on pathology) has varied from study to study generally in the 45–60% range. In our study, we found less than 10% viable cells in only 35% of our YA specimen after neoadjuvant chemotherapy, compared to 55% in our pediatric specimen. Although this trend was not statistically significant, it may suggest that YA osteosarcoma is more chemotherapy-resistant and should be investigated in other studies. Lastly, more YA patients progressed on primary therapy, which could also indicate more resistant disease biology, less effective chemotherapy delivery to the tumor secondary to pharmacologic handling of the agents, or less frequent dosing of chemotherapy.

All patients who had recurrent disease received chemotherapy, precluding us from comparing this subset to a subset that did not receive chemotherapy for recurrence. We did, however, determine that, of the YA patients who had recurrence, the median postrelapse survival (PRS) was 1.5 years. In a recent report, the use of chemotherapy demonstrated a trend toward improved postrelapse event-free but not overall survival for patients who did not achieve a second complete remission; however, chemotherapy was also associated with a worse overall outcome [34]. Other studies have demonstrated improved survival with the use of chemotherapy for tumors that are not completely resectable [35–37]. Trials with clear endpoints based on good historical data or more difficult to perform randomized, prospective trials are needed to further characterize the association between chemotherapy and improved survival following relapse.

As evidenced in this study, the lack of consensus on a YA chemotherapy protocol resulted in a variety of regimens being utilized at a single center by multiple oncologists, likely reflecting national practice. The COG experience found that 12% of patients were in the YA range, an underrepresentation based on incidence data [14]. Certainly the best way to learn about this patient population would be through active clinical trial participation. While lacking the power of consortium data to detect differences, our study provides detailed chemotherapeutic delivery data over a time period that spans multiple medical and pediatric oncologists who cared for YA osteosarcoma patients. Given the paucity of YA patients who enroll on clinical trials, this may serve as a baseline for future studies. Because our MAP+ YA cohort demonstrated trends toward improved survival, continuing to enroll YA patients on clinical trials should be encouraged when possible. The inability of adult centers to participate as COG centers has limited clinical trial availability for YA patients in our area. Concerted efforts are ongoing to address these organizational barriers to YA patient enrollment [38].

The limitations of this study stem from the nature of treating a rare disease and its retrospective nature. The small sample size limited the power of this study, the use of nonstandardized clinical protocols across patients, and the incomplete records in the pediatric cohort likely limit the generalizability of our results. Nonetheless, this work is consistent with other studies that have demonstrated poorer outcomes for osteosarcoma patients over age 18. We found that most presenting factors, including factors with strong prognostic implications, were not distinct between the pediatric and YA cohorts and likely do not contribute to the discrepancy in outcomes that we observed. Although the study did not have the power to detect significance, the trends in our data may indicate that YA patients may have more chemotherapy-resistant disease, as suggested by progression on therapy and fewer cases of good histologic response after neoadjuvant therapy. We also observed that a marked number of YA patients do not achieve the cumulative chemotherapy doses commonly used to treat pediatric patients, which may contribute to the poorer outcomes of this population.

## Figures and Tables

**Table 1:** Study population characteristics.

	Pediatric cohort		YA cohort		*P* value
	*N *	%	*N *	%	
Gender					
Male	18	55	23	64	0.47
Female	15	45	13	36	
Location					
Long bones	32	97	29	81	0.13
Pelvic	0	0	3	8	
Other	1	3	4	11	
Histology					
Osteoblastic	24	73	21	58	0.28
Fibroblastic	2	6	3	8	
Chondroblastic	4	12	8	22	
Giant Cell	0	0	2	6	
Telangiectatic	2	6	0	0	
Extraskeletal	0	0	1	3	
Periosteal	0	0	1	3	
Missing	1	3	0	0	
Size					
<8 cm	15	46	12	33	0.21
>8 cm	14	42	22	61	
Missing	4	12	2	6	
Pathological fracture					
Yes	6	18	3	8	0.29
No	27	82	33	92	

**Table 2:** Therapeutic modalities used.

	Pediatric cohort		YA cohort		*P* value
	*N *	%	*N *	%	
Surgery					
Yes	33	100	34	94	0.49
No	0	0	2	6	
Type of surgery					
Limb salvage	31	94	30	88	0.67
Amputation	2	6	4	12	
Margins					
Positive	1	3	1	3	0.86
Negative	24	73	31	91	
Missing	8	24	2	6	
Chemotherapy					
Yes	33	100	36	100	1.00
No	0	0	0	0	
Histologic response to chemotherapy					
Good	18	55	12	35	0.06
Poor	8	24	16	47	
No neoadj chemo	1	3	5	15	
Missing	6	18	1	3	
Radiation therapy					
Yes	4	12	5	14	1.00
No	29	85	31	86	

**Table 3:** Therapies used for patients with recurrence in the YA cohort without metastatic disease at diagnosis.

Disease Description	Primary therapy received	Childhood MAP regimen achieved?	Histological response to primary therapy	Progression on primary therapy	Secondary therapy received	Progression on subsequent therapy	Time to recurrence in years	Type of recurrence	Survival time in years	Current status
Chondroblastic osteosarcoma of the pelvis	MAPIE, proton beam therapy	No	N/A	No	Gem, Tax, wedge resection, MTP-PE, bevacizumab, zoledronic acid	Yes	0.31	(1) Lung; (2) local progression with chest wall metastasis	2.37	DOD

Osteoblastic osteosarcoma of the distal femur	Neoadj/adj MAPI, resection	Yes	Poor	No	Inhaled chemotherapy, vaccine trial	Yes	1.10	(1) Lung, lymph nodes; (2) apex of heart, mitral valve	2.63	DOD

Osteoblastic osteosarcoma of proximal fibula	Neoadj/adj MAPIE, resection	Yes	Unknown	No	Amputation, serial lung excisions, IEP, trimetrexate trial	Yes	1.60	(1) Local; (2) multiple lung nodules bilaterally	3.98	DOD

Osteoblastic osteosarcoma of distal femur	VP-16, IE, AP, resection	No	Good	No	MAP, amputation, gem, phase I ASAP, phase I Ski-606	Yes	0.20	(1) Local; (2) lung, hip, and pelvis	4.05	DOD

Osteosarcoma of unknown histology in proximal humerus	Neoadj/adj MAP, forequarter amputation	No	Poor	Yes	IE	Yes	0.14	Lung	1.84	DOD

Osteoblastic osteosarcoma of distal tibia	Neoadj/adj MAP, Resection	Yes	Good	No	Wedge resection, IE	No	0.87	Lung	3.18	NED

Chondroblastic osteosarcoma of the chest wall	Neoadj/adj MAP, resection	No	Good	Yes	Unk	Unk	0.50	Local	3.18	Unk

Chondroblastic osteosarcoma of the chest wall	Resection, adj MAP	No	N/A	No	AI, thoracotomy, neurosurgery, neuroradiation	Yes	1.46	(1) Local; (2) lung and brain	4.30	DOD

Osteoblastic osteosarcoma of the proximal tibia	Resection, Adj AP	No	N/A	No	AI, Above-knee amputation, AP, wedge resection, rapamycin	Yes	0.61	(1) Local; (2) proximal fibula; (3) lung	2.90	DOD

Osteosarcoma unclassifiable subtype, pelvis	MAID, radiation therapy	No	N/A	Yes	AP	Yes	0.00	Spine and femoral head	1.15	DOD

Osteoblastic osteosarcoma of proximal tibia	Neoadj/adj MAI, resection	No	Poor	No	Gem, Tax, imatinib, P, Doxil, local resection, wedge resection	Yes	0.33	(1) Lung; (2) local	2.35	DOD

Periosteal osteosarcoma of proximal tibia	Neoadj MAP, resection	No	Poor	No	Local radiation, resection, IE, wedge resection	No	1.08	(1) Local, (2) lung	4.80	NED

Giant cell osteosarcoma in sacral spine	MAP, radiation therapy, IE, temozolomide	No	N/A	Yes	Temozolomide radiation, IE, denosumab, pazopanib, palliative surgery	Yes	1.42	(1) Local; (2) rib; (3) acetabulum	3.38	AWD

M: methotrexate; A: doxorubicin; P: cisplatin; I: ifosfamide; E: etoposide; gem: gemcitabine; Tax: Taxotere; MTP-PE: mifamurtide; Unk: unknown; Neoadj: neoadjuvant; Adj: adjuvant; NED: no evidence of disease; DOD: died of disease; AWD: alive with disease. Time to recurrence was measured from end therapy date to date of recurrence. Survival time was measured from date of diagnosis to date of death or censored date. Recurrences are numbered in the order of discovery.
